# Pregnane X Receptor Mediates Atherosclerosis Induced by Dicyclohexyl Phthalate in LDL Receptor-Deficient Mice

**DOI:** 10.3390/cells11071125

**Published:** 2022-03-26

**Authors:** Jingwei Liu, Rebecca Hernandez, Xiuchun Li, Zhaojie Meng, Hong Chen, Changcheng Zhou

**Affiliations:** 1 Division of Biomedical Sciences, School of Medicine, University of California, Riverside, CA 92521, USA; jingwei.liu@medsch.ucr.edu (J.L.); rebecca.hernandez@medsch.ucr.edu (R.H.); xiuchun.li@medsch.ucr.edu (X.L.); mengzj1616@gmail.com (Z.M.); 2 Department of Surgery, Vascular Biology Program, Harvard Medical School, Boston Children’s Hospital, Boston, MA 02115, USA; hong.chen@childrens.harvard.edu

**Keywords:** endocrine disrupting chemicals, atherosclerosis, phthalate, plastics, pregnane X receptor

## Abstract

Plastic-associated endocrine disrupting chemicals (EDCs) have been implicated in the etiology of cardiovascular disease (CVD) in humans, but the underlying mechanisms remain elusive. Dicyclohexyl phthalate (DCHP) is a widely used phthalate plasticizer; whether and how exposure to DCHP elicits adverse effects in vivo is mostly unknown. We previously reported that DCHP is a potent ligand of the pregnane X receptor (PXR) which acts as a xenobiotic sensor to regulate xenobiotic metabolism. PXR also functions in macrophages to regulate atherosclerosis development in animal models. In the current study, LDL receptor-deficient mice with myeloid-specific PXR deficiency (PXR^ΔMye^LDLR^−/−^) and their control littermates (PXR^F/F^LDLR^−/−^) were used to determine the impact of DCHP exposure on macrophage function and atherosclerosis. Chronic exposure to DCHP significantly increased atherosclerotic lesion area in the aortic root and brachiocephalic artery of PXR^F/F^LDLR^−/−^ mice by 65% and 77%, respectively. By contrast, DCHP did not affect atherosclerosis development in PXR^ΔMye^LDLR^−/−^ mice. Exposure to DCHP led to elevated expression of the scavenger receptor CD36 in macrophages and increased macrophage form cell formation in PXR^F/F^LDLR^−/−^ mice. Our findings provide potential mechanisms underlying phthalate-associated CVD risk and will ultimately stimulate further investigations and mitigation of the adverse effects of plastic-associated EDCs on CVD risk in humans.

## 1. Introduction

Atherosclerotic cardiovascular disease (CVD) is the leading cause of global morbidity and mortality [1,2]. In addition to the well-known contributing factors including unhealthy diet and sedentary lifestyles [3,4], exposure to environmental chemicals such as endocrine disrupting chemicals (EDCs) has been implicated in the etiology of CVD [2,5,6,7]. For example, the ubiquitous plastic-associated EDCs including base chemical bisphenol A (BPA) and numerous plasticizers have been associated with increased CVD risk in humans [2,5,6,7,8,9,10,11,12,13]. While BPA has attracted considerable attention and controversy, many other plastic-associated EDCs such as phthalates are produced in high volume and can also cause adverse effects on cardiovascular health in the general population [6,7,11,14,15,16]. However, the mechanisms by which exposure to these EDCs influences CVD risk are still poorly understood, which continues to hamper rational assessment of the health risks of EDC exposure.

To sense and respond to environmental chemicals, mammals have evolved a defensive network governed by xenobiotic receptors, such as pregnane X receptor (PXR) [17,18,19,20]. PXR is a nuclear receptor that regulates many genes involved in xenobiotic metabolism [17,18,19]. Interestingly, many plastic-associated EDCs including BPA, phthalate plasticizers, and phthalate substitutes have been identified as potent agonists for PXR [2,20,21,22,23,24]. For example, we found that BPA and its analogs such as bisphenol B (BPB) and 4-cumylphenol (HPP) can strongly activate human PXR [21]. Many plasticizers, including phthalates (e.g., Di(2-ethylhexyl) phthalate (DEHP)) and phthalate substitutes (e.g., Tributyl citrate (TBC)), are also PXR ligands [22,23]. 

In addition to functioning as a xenobiotic sensor, PXR has recently been revealed to play important roles in regulating lipid homeostasis and atherogenesis [2,20,23,25,26,27,28,29,30,31,32,33,34,35,36]. Activation of PXR by various ligands including EDCs have been demonstrated to cause adverse effects on hepatic or intestinal lipid homeostasis in wild-type mice, leading to increased plasma cholesterol levels and atherogenic lipoprotein LDL levels [23,26,28,31,34,35,36]. In addition to liver and intestine, essential for lipid homeostasis, PXR is also expressed in immune cells including macrophages [26,37,38,39,40,41,42] which play a key role in atherosclerosis development [43,44]. We previously demonstrated that PXR can regulate macrophage functions to promote atherosclerosis development in apolipoprotein E-deficient (ApoE^−/−^) mice [25,26,32,33] as well as LDL receptor-deficient (LDLR^−/−^) mice [45]. More recently, we identified a widely used phthalate, dicyclohexyl phthalate (DCHP), as a ligand for PXR and short-term exposure to DCHP led to dysregulated lipid homeostasis [24]. However, it is unknown whether chronic DCHP exposure can also affect macrophage functions to regulate atherogenesis in appropriate animal models.

In the current study, we used a myeloid-specific PXR-deficient LDLR^−/−^ mouse model to investigate the impact of chronic DCHP exposure on macrophage functions and atherosclerosis development. We demonstrate, for the first time to our knowledge, that chronic exposure to DCHP increased atherosclerosis in LDLR^−/−^ mice, and deficiency of myeloid PXR protected mice from DCHP-induced atherosclerosis. 

## 2. Materials and Methods

### 2.1. Animals and Treatment

To study the function of PXR in macrophages, we previously generated myeloid-specific PXR-deficient (PXR^ΔMye^) mice on C57BL/6 background by crossing mice carrying loxP-flanked PXR alleles (PXR^F/F^) [34,45] with LysM-Cre transgenic mice [46], as we previously described [45]. To increase susceptibility to atherosclerotic development, the PXR^ΔMye^ mice were then crossed with LDLR^−/−^ mice to generate PXR^ΔMye^LDLR^−/−^ and PXR^F/F^LDLR^−/−^ mice [45]. All mice used in this study had PXR^F/F^LDLR^−/−^ double-mutant background, and PXR^ΔMye^LDLR^−/−^ mice carried heterozygous knock-in for LysM-Cre. 

To investigate the effects of DCHP exposure on atherosclerosis development, 4-week-old male PXR^ΔMye^LDLR^−/−^ and PXR^F/F^LDLR^−/−^ littermates were fed ad libitum on a semisynthetic low-fat AIN76 diet (4.2% fat and 0.02% cholesterol; Research Diet) [25,45,47,48,49] and were also treated with 10 mg/kg body weight of DCHP (Sigma-Aldrich) or vehicle control (corn oil) daily by oral gavage for 12 weeks until euthanasia at 16 weeks of age. All mice were housed in pathogen-free microisolator cages in a temperature-controlled room with a 12 h light/dark cycle. Body weight was measured weekly and intraperitoneal glucose tolerance test (GTT) was performed as previously described [48,49,50]. On the day of euthanasia, mice were fasted for 6 hr following the dark cycle, and blood was collected as previously described [35,51,52]. The major organs/tissues (e.g., liver, kidney, subcutaneous and epidydimal white adipose tissue, and brown adipose tissue) were collected and weighed as previously described [50,53,54]. The animal studies were performed in compliance with approved protocols by the Institutional Animal Care and Use Committee of the University of California, Riverside.

### 2.2. Blood Analysis

The blood samples were collected by left ventricle puncture and centrifuged at 1500× *g* for 15 min at 4 °C to collect the serum. The top clear phase was collected for lipid analysis. Total cholesterol and triglyceride concentrations in serum were analyzed using the Wako Cholesterol E enzymatic colorimetric assay (999-02601) and the Wako L-Type TG M assay (994-02891) kits according to the manufacture’s instruction (FUJIFILM Medical Systems U.S.A., Inc., Richmond, VA, USA). 

### 2.3. Atherosclerotic Lesion Analysis

At the end of the study, atherosclerotic lesions were analyzed at the aortic root and the brachiocephalic artery of PXR^ΔMye^LDLR^−/−^ and PXR^F/F^LDLR^−/−^ mice. To analyze the lesion areas at the aortic root, Optimal Cutting Temperature (OCT)-compound-embedded heart samples were sectioned at a 12 µm thickness and all the three valves of the aortic root in the same plane were kept as described previously [26,32,33,49,51]. Sections were then stained with Oil-red-O. To analyze the atherosclerotic lesions at the brachiocephalic artery, the OCT-embedded brachiocephalic arteries were sectioned at a thickness of 10 µm from distal to proximal. Sections were also stained with oil red O, and atherosclerotic lesions were then quantified in three equidistant stained sections 200, 400, and 600 µm proximal from the branching point of the brachiocephalic artery into the carotid and subclavian arteries [49,51].

### 2.4. Isolation of Primary Macrophages and Analysis of Their Related Functions

Primary peritoneal macrophages were isolated from PXR^ΔMye^LDLR^−/−^ and PXR^F/F^LDLR^−/−^ mice as previously described [51,55]. The freshly isolated macrophages were attached to coverslips for 4 h, and then stained with Oil-red-O and hematoxylin. Macrophages containing lipid droplets (>10) were then counted as foam cells. For the macrophage adhesion assay, peritoneal macrophages were first labeled with calcein acetoxymethyl and were then incubated with primary porcine endothelial cells [51,55]. The attached macrophages were fixed and counted under microscope. For the macrophage migration assay, transwells with 8-μm pore polycarbonate membrane inserts (Corning, Glendale, AZ, USA) were used. Macrophages with were seeded on the transwell filters with serum-free MEM media (Corning, Glendale, AZ, USA). The lower chambers were filled with the complete MEM media (Corning, Glendale, AZ, USA) containing 10% fetal bovine serum (FBS) as a chemoattractant. After incubation for 16 h, macrophages were removed from the upper surface of the insert by using Q-Tips. The membranes were fixed with 4% paraformaldehyde (PFA) and were stained with hematoxylin (Leica, Wetzlar, Germany). The membranes were then mounted on the slides by using glycerol gelatin. The hematoxylin-stained macrophages were counted by using a microscope. For the macrophage lipid uptake assay, macrophages were incubated with serum-free media containing 100 μg/mL of oxidized LDL (Athens Research & Technology, Athens, GA, USA) for 24 h. The cells were then stained with Oil-red-O and hematoxylin. Macrophages containing lipid droplets (>10) were then counted as foam cells. 

### 2.5. RNA Isolation and Quantitative Real-Time PCR Analysis

TRIzol reagent (Thermo Fisher Scientific, Carlsbad, CA, USA) was used for isolating total RNA from mouse tissues or cells. Quantitative real-time PCR (QPCR) was performed by using gene-specific primers and the SYBR Green PCR kit (Bio-Rad, Hercules, CA, USA) on a CFX Real-Time PCR Instrument (Bio-Rad, Hercules, CA, USA) as previously described [24,35,52]. The sequences of primer sets used in this study are listed in Supplemental Table S1.

### 2.6. Immunohistochemistry

Immunohistochemistry was performed on 12-μm OCT-embedded aortic root sections. The slides were first fixed in 4% PFA for 15 min and permeabilized with PBS containing 0.1% Triton X-100 (PBST) for 15 min. The slides were then blocked by PBST containing 5% bovine serum albumin (BSA) (Sigma-Aldrich, St. Louis, MO, USA) for 1 h at room temperature. For the immunostaining, the sections were incubated with rat anti-mouse CD68 antibodies (1:100; AbD Serotec, Oxford, UK) or rat anti-mouse CD36 antibodies (1:100; AbD Serotec, Oxford, UK). After overnight incubation at 4 °C, the slides were rinsed with PBS and then incubated with secondary antibodies (1:500; Life Technologies, Carlsbad, CA, USA). The nuclei were also stained by mounting the slides with 4’,6-diamidino-2-phenylindole (DAPI) medium (Vector Laboratories, Burlingame, CA, USA). Images were then taken by using a Nikon fluorescence microscope (Nikon, Melville, NY, USA). 

### 2.7. Statistical Analysis

All data are presented as the mean ± SEM and N numbers are listed in the figure legends. Individual pairwise comparisons were analyzed by two-sample, two-tailed Student’s t-test. Two-way ANOVA was used when multiple comparisons were made, followed by a Bonferroni multiple comparisons test. The analyses were performed using GraphPad Prism. *p* < 0.05 was considered statistically significant. 

## 3. Results

### 3.1. Exposure to DCHP Does Not Affect Metabolic Phenotypes and Lipid Profiles of PXR^F/F^ LDLR^−/−^ and PXR^ΔMye^LDLR^−/−^ Mice

To study the functions of macrophage PXR in atherosclerosis, we previously generated LDLR^−/−^ mice with myeloid-specific PXR deficiency (PXR^ΔMye^LDLR^−/−^) mice by crossing PXR^ΔMye^ (LysM-Cre/PXR^F/F^) mice with LDLR^−/−^ mice [45]. To determine whether DCHP affect macrophage PXR signaling to influence atherosclerosis development, 4-week-old male PXR^ΔMye^LDLR^−/−^ and PXR^F/F^LDLR^−/−^ littermates were treated with 10 mg/kg body weight of DCHP or vehicle control by daily oral gavage for 12 weeks. The mice used in this study had PXR^F/F^LDLR^−/−^ double-mutant background, and PXR^ΔMye^LDLR^−/−^ mice also carried heterozygous knock-in for LysM-Cre. PXR^ΔMye^LDLR^−/−^ and PXR^F/F^LDLR^−/−^ mice were fed a low-fat AIN76 diet containing 4.3% fat and 0.02% cholesterol [47]. We and others have successfully used this diet to induce atherosclerosis without eliciting obesity and associated metabolic disorders in LDLR^−/−^ or ApoE^−/−^ mice [25,45,47,48,49,56]. 

**Figure 1:**
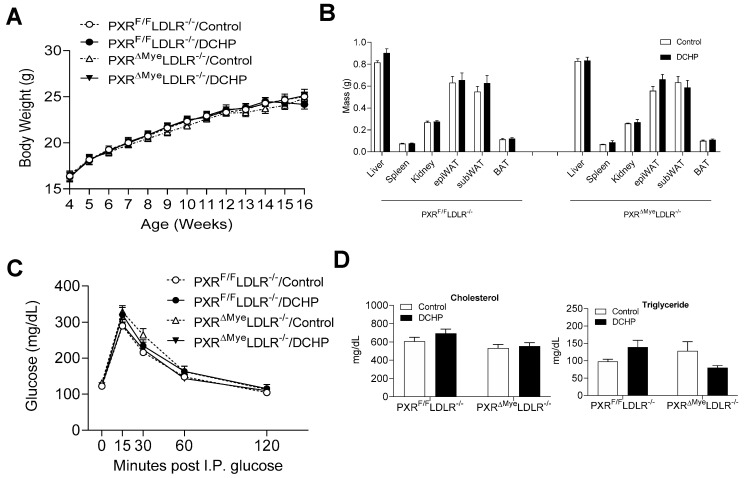
DCHP exposure does not affect metabolic phenotypes and plasma lipid profiles in LDLR^−/−^ mice. Four-week-old male PXR^F/F^LDLR^−/−^ and PXR^ΔMye^LDLR^−/−^ littermates were treated with 10 mg/kg/day of DCHP or vehicle control daily by oral gavage for 12 weeks. Growth curve (**A**), major organ weight (**B**), glucose tolerance (**C**), and serum cholesterol and triglyceride levels (**D**) were measured. Data are represented as mean ± SEM (n = 6–12). subWAT, subcutaneous white adipose tissue; epiWAT, epididymal white adipose tissue; and BAT, brown adipose tissue.

Exposure to DCHP did not affect body weight and growth curve of PXR^ΔMye^LDLR^−/−^ and PXR^F/F^LDLR^−/−^ mice (Figure 1A). These mice also had similar organ weights which were not affected by DCHP treatment (Figure 1B). Glucose tolerance tests also demonstrated that myeloid PXR-deficiency or DCHP exposure did not affect glucose tolerance in either PXR^F/F^LDLR^−/−^ or PXR^ΔMye^LDLR^−/−^ mice (Figure 1C). Next, we measured the plasma lipid levels and found that exposure to DCHP did not affect plasma cholesterol and triglyceride levels in PXR^F/F^LDLR^−/−^ and PXR^ΔMye^LDLR^−/−^ mice (Figure 1D). 

### 3.2. Chronic Exposure to DCHP Leads to Increased Atherosclerosis in PXR^F/F^ LDLR^−/−^ but Not PXR^ΔMye^LDLR^−/−^ Mice

**Figure 2:**
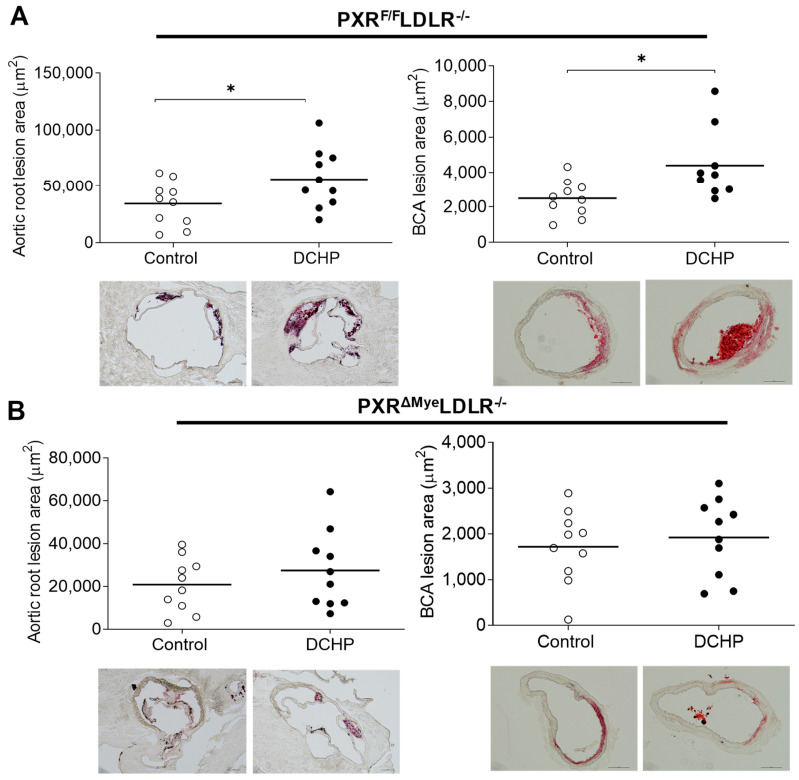
Exposure to DCHP leads to increased atherosclerosis in PXR^F/F^LDLR^−/−^ but not PXR^ΔMye^LDLR^−/−^ mice. Four-week-old male PXR^F/F^LDLR^−/−^ and PXR^ΔMye^LDLR^−/−^ littermates were treated with 10 mg/kg/day of DCHP or vehicle control daily by oral gavage for 12 weeks. Quantitative analysis of atherosclerotic lesion size in the aortic root and brachiocephalic artery (BCA) of PXR^F/F^LDLR^−/−^ (**A**) and PXR^ΔMye^LDLR^−/−^ (**B**) mice (n = 9–10 per group, * *p* < 0.05). Representative Oil red O-stained sections are shown as indicated.

Atherosclerotic lesion areas were then analyzed in the aortic root and brachiocephalic artery (BCA) as shown in Figure 2. We found that DCHP exposure significantly increased atherosclerotic lesion areas by 65% in the aortic root of PXR^F/F^LDLR^−/−^ mice (56,564 ± 8295 μm^2^ vs. 34,290 ± 6160 μm^2^) (Figure 2A, left panel). Consistently, exposure to DCHP also increased the atherosclerotic lesion areas in the BCA of PXR^F/F^LDLR^−/−^ mice by 77% (4399 ± 715 μm^2^ vs. 2481 ± 338 μm^2^) (Figure 2A, right panel). By contrast, exposure to DCHP did not affect atherosclerotic lesion areas in the aortic root or BCA of PXR^ΔMye^LDLR^−/−^ mice (Figure 2B). Collectively, these results suggest that exposure to DCHP increases atherosclerosis development in LDLR^−/−^ mice, and that myeloid PXR signaling contributes to DCHP’s atherogenic effects in vivo.

### 3.3. DCHP Exposure Increases Lipid Accumulation and Foam Cell Formation in Macrophages of PXR^F/F^LDLR^−/−^ Mice

**Figure 3:**
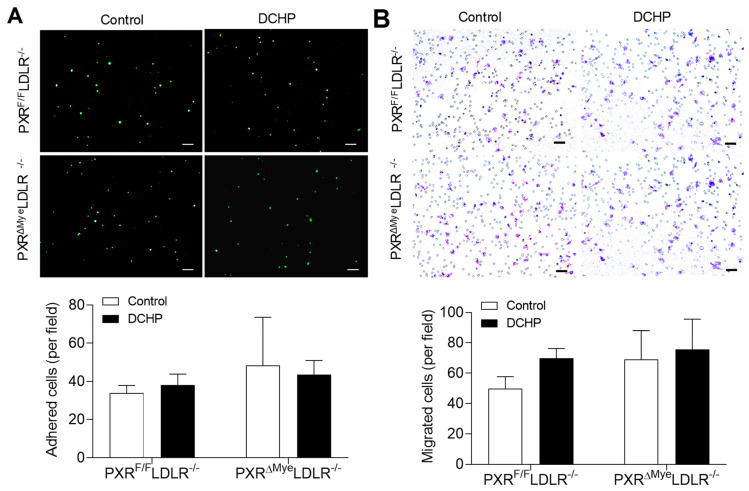
DCHP exposure does not affect the migration and adhesion properties of macrophages of PXR^F/F^LDLR^−/−^ and PXR^ΔMye^LDLR^−/−^ mice. Four-week-old male PXR^F/F^LDLR^−/−^ and PXR^ΔMye^LDLR^−/−^ littermates were treated with 10 mg/kg/day of DCHP or vehicle control daily by oral gavage for 12 weeks. (**A**) Peritoneal macrophages isolated from the PXR^F/F^LDLR^−/−^ and PXR^ΔMye^LDLR^−/−^ mice were labeled with calcein acetoxymethyl and cultured with primary porcine endothelial cell monolayer for 30 min. Adhered cells were then counted under a fluorescence microscope (scale bars = 100 µm). (**B**) Peritoneal macrophages isolated from the PXR^F/F^LDLR^−/−^ and PXR^ΔMye^LDLR^−/−^ mice were seeded on the Matrigel-coated transwell filters. Cells that infiltrated and migrated to the underside of the transwell were stained with hematoxylin and counted under the microscope (scale bars = 100 µm). Quantitative analysis of the migrated and adhered cells was displayed below the representative images. Data are represented as mean ± SEM (n = 3).

Macrophages play an important role in atherogenesis and accumulation of lipid-loaded macrophages is considered as non-negligible feature of atherosclerosis [43]. To investigate whether DCHP exposure affects macrophage functions related to atherosclerosis development, we first examined the impact of DCHP treatment on macrophage adhesion and migration properties. Peritoneal macrophages were isolated from the PXR^F/F^LDLR^−/−^ and PXR^ΔMye^LDLR^−/−^ mice exposed to DCHP or vehicle control. Incubation of freshly isolated peritoneal macrophages with primary endothelial cells (ECs) showed that exposure to DCHP did not affect adhesion of control of PXR-deficient macrophages to ECs (Figure 3A). We also investigated the effects of DCHP exposure on macrophage migration by transwell assay. As shown in Figure 3B, exposure of DCHP did not affect the migration ability of either control or PXR-deficient macrophages. 

**Figure 4:**
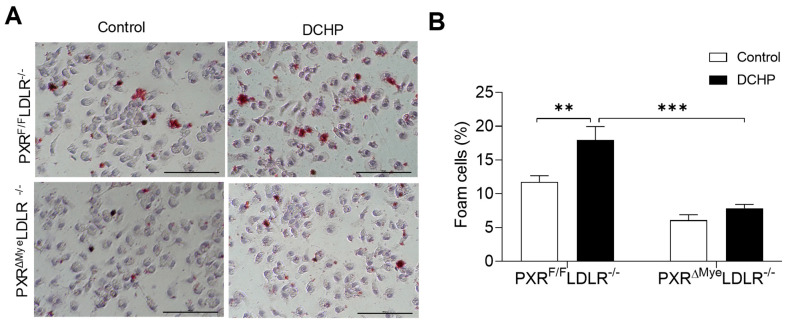
Deficiency of PXR reduces DCHP-induced macrophage foam cell formation. Four-week-old male PXR^F/F^LDLR^−/−^ and PXR^ΔMye^LDLR^−/−^ littermates were treated with 10 mg/kg/day of DCHP or vehicle control daily by oral gavage for 12 weeks. (**A**) Peritoneal macrophages were isolated and stained with Oil Red O and hematoxylin (scale bars = 100 µm), (**B**) Foam cell quantification from peritoneal macrophages in studies described in panel A. Data are represented as mean ± SEM (n = 3, ** *p* < 0.01, and *** *p* < 0.001).

**Figure 5:**
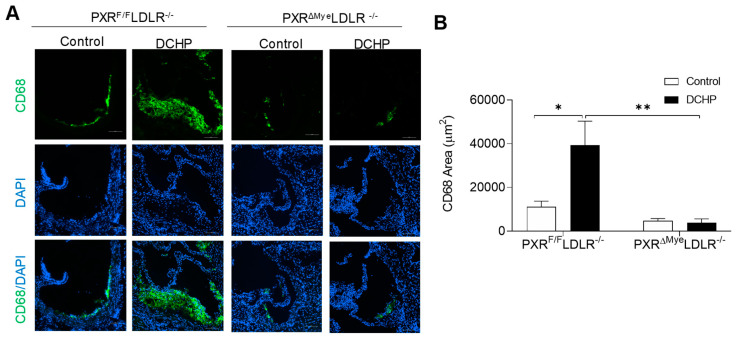
Exposure to DCHP increases atherosclerotic lesional macrophage content in PXR^F/F^LDLR^−/−^ but not PXR^ΔMye^LDLR^−/−^ mice. Four-week-old male PXR^F/F^LDLR^−/−^ and PXR^ΔMye^LDLR^−/−^ littermates were fed a low-fat diet and treated by oral gavage with 10 mg/kg body weight of vehicle (corn oil) or DCHP daily for 12 weeks. (**A**) Representative images of immunofluorescence staining of CD68 (Green) in the aortic root of PXR^F/F^LDLR^−/−^ and PXR^ΔMye^LDLR^−/−^ mice (Scale bar = 100 µm). The nuclei were stained with DAPI (Blue). (**B**) Quantification analysis of CD68 staining area in the aortic root of PXR^F/F^LDLR^−/−^ and PXR^ΔMye^LDLR^−/−^ mice. Data are represented as mean ± SEM (n = 5, * *p* < 0.05 and ** *p* < 0.01).

We previously reported that activation of PXR can induce lipid accumulation in the macrophages of ApoE^−/−^ mice, which contributes to PXR ligands’ pro-atherogenic effects [26,32]. We then performed Oil red O staining to assess neutral lipid levels in fresh isolated peritoneal macrophages of PXR^F/F^LDLR^−/−^ and PXR^ΔMye^LDLR^−/−^ mice. Exposure to DCHP promoted lipid accumulation and foam cell formation in macrophages of PXR^F/F^LDLR^−/−^ mice, but not in that of PXR^ΔMye^LDLR^−/−^ mice (Figure 4). Consistent with macrophage foam cell formation results, immunostaining for macrophage marker demonstrated that exposure to DCHP led to increased macrophage content in atherosclerotic lesions of PXR^F/F^LDLR^−/−^ mice, but not in that of PXR^ΔMye^LDLR^−/−^ mice (Figure 5). Thus, PXR signaling mediates DCHP-stimulated macrophage lipid accumulation and foam cell formation in LDLR^−/−^ mice. 

### 3.4. DCHP-Mediated PXR Activation Stimulates Macrophage CD36 Expression and Increases Lipid Uptake by Macrophages of PXR^F/F^LDLR^−/−^ Mice

**Figure 6:**
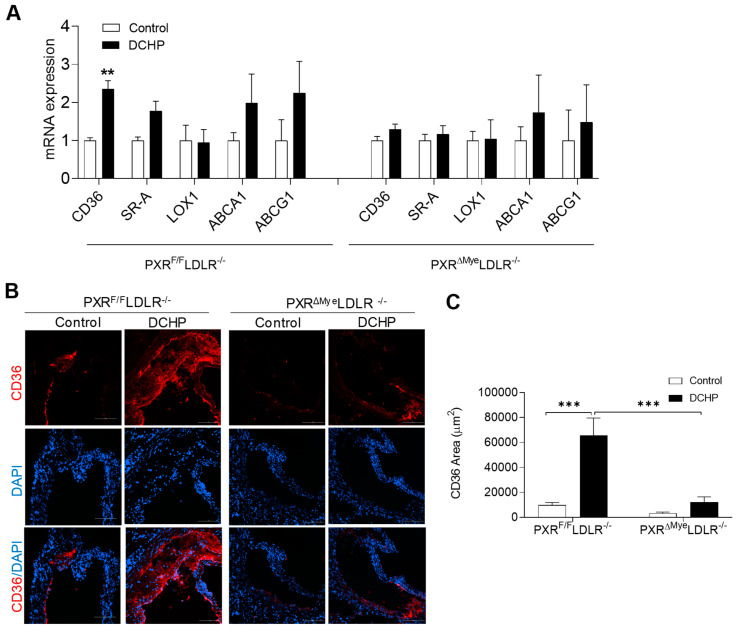
Deficiency of myeloid PXR reduces DCHP-induced CD36 expression in macrophages and atherosclerotic lesions of PXR^ΔMye^LDLR^−/−^ mice. Four-week-old male PXR^F/F^LDLR^−/−^ and PXR^ΔMye^LDLR^−/−^ littermates were fed a low-fat diet and treated by oral gavage with 10 mg/kg body weight of DCHP or vehicle control daily for 12 weeks. (**A**) Total RNAs were isolated from fresh isolated peritoneal macrophages of PXR^F/F^LDLR^−/−^ and PXR^ΔMye^LDLR^−/−^ mice, and the expression levels of indicated genes were analyzed by QPCR (n = 4–6, ** *p* < 0.01). (**B**) Representative images of immunofluorescence staining of CD36 (Red) in the aortic root of PXR^F/F^LDLR^−/−^ and PXR^ΔMye^LDLR^−/−^ mice (Scale bar = 100 µm). The nuclei were stained with DAPI (Blue). (**C**) Quantification analysis of CD36 staining area in the aortic root of PXR^F/F^LDLR^−/−^ and PXR^ΔMye^LDLR^−/−^ mice (n = 5, *** *p* < 0.001). Data are represented as mean ± SEM.

We previously demonstrated that PXR can regulate the expression of CD36 in macrophages, a key macrophage lipogenic gene, to affect macrophage lipid uptake foam cell formation [26,32,45]. CD36 is a scavenger receptor that plays a key role in macrophage lipid uptake and foam cell formation [20,57,58]. Indeed, we found that DCHP exposure led to significantly increased CD36 expression in macrophages of PXR^F/F^LDLR^−/−^ mice, but not that of PXR^ΔMye^LDLR^−/−^ mice. By contrast, the expression of other scavenger receptors, SR-A and LOX-1, as well as ABC transporters, ABCA1 and ABCG1, were not significantly affected by DCHP exposure in macrophages of PXR^F/F^LDLR^−/−^ and PXR^ΔMye^LDLR^−/−^ mice (Figure 6A). Consistent with the macrophage results, analysis of atherosclerotic lesions confirmed that DCHP exposure significantly increased CD36 content in the plaques of PXR^F/F^LDLR^−/−^ mice, but had no effects on CD36 content in PXR^ΔMye^LDLR^−/−^ mice (Figure 6B,C). 

**Figure 7:**
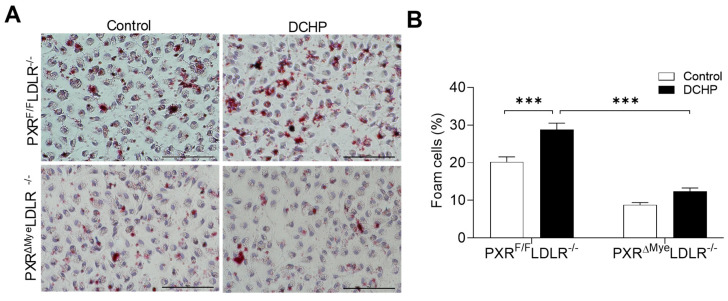
Ablation of PXR ameliorates DCHP-elicited lipid uptake by macrophages. Four-week-old male PXR^F/F^LDLR^−/−^ and PXR^ΔMye^LDLR^−/−^ littermates were fed a low-fat diet and treated by oral gavage with 10 mg/kg body weight of DCHP or vehicle control daily for 12 weeks. (**A**) Peritoneal macrophages were isolated and incubated with oxLDL (200 µg/mL) for 24 h and stained with Oil Red O and hematoxylin (scale bars = 100 µm). (**B**) Foam cell quantification from peritoneal macrophages in studies described in panel A. Data are represented as mean ± SEM (n = 3, *** *p* < 0.001).

Previous studies have demonstrated that CD36 mediates the uptake of oxidized LDL (oxLDL), the important atherogenic LDL, by macrophages [57,58]. We then performed lipid uptake assays in control and PXR-deficient macrophages using oxLDL. Consistent with CD36 expression results, DCHP exposure led to significantly increased oxLDL uptake in macrophages of PXR^F/F^LDLR^−/−^ mice, but deficiency of PXR abolished DCHP-stimulated oxLDL uptake in macrophages of PXR^ΔMye^LDLR^−/−^ mice (Figure 7). Therefore, DCHP-stimulated CD36 expression in macrophage likely contributes to increased foam cell formation and atherosclerosis development in PXR^F/F^LDLR^−/−^ mice. 

## 4. Discussion

Plastics have become an essential part of modern society due to their durability, versatility, and low cost of fabrication. The annual global plastic production has increased nearly 200 fold, from 2 million to 380 million metric tons during 1950 to 2015, and has been predicted to reach 1.1 billion metric tons in 2050 [59,60]. Plastic pollution has prompted considerable environmental and public health concerns [61,62,63]. Phthalates are used as plasticizers to make numerous products and human exposure to these chemicals is ubiquitous. The potential adverse health outcomes induced by phthalates exposure include declined reproductive capacity [64,65] and increased risks of cancer [66,67,68], diabetes [69,70], obesity [71,72], and CVD [73,74,75,76]. However, existing evidence is predominantly obtained from research data on a few specific phthalates such as DEHP [77,78]. By contrast, little is known about the adverse effects of several other widely used phthalates including DCHP. Thus, DCHP has recently been designated by the EPA as a high-priority substance for risk evaluation [79]. We recently identified DCHP as a ligand of PXR which has pro-atherogenic properties in animal models [24]. In the current study, we report that chronic DCHP exposure increased atherosclerosis development in LDLR^−/−^ mice in a PXR-dependent manner. Exposure to DCHP significantly increased atherosclerotic lesion size in the aortic root and BCA of PXR^F/F^LDLR^−/−^, but not PXR^ΔMye^LDLR^−/−^ mice. DCHP-mediated PXR activation led to increased macrophage lipid uptake and foam cell formation, which likely contribute to the increased atherosclerosis development in those mice. To our knowledge, our study is the first to demonstrate the impact of DCHP exposure on the development of atherosclerosis in an appropriate small animal model.

Exposure to phthalates may occur through multiple routes, including ingestion (e.g., from food packaging and children’s toys), inhalation (e.g., from building materials and furniture), and dermal contact (e.g., cosmetics and other personal care products) [80,81]. Dietary intake has been considered as the major route of exposure to phthalates [82]. Therefore, mice were exposed to DCHP via daily oral gavage in the current study. Humans can be exposed to phthalates at relatively high levels [15,83,84,85,86,87,88,89] and DCHP can be found in consumer products, foods, and environmental samples including water and indoor particulate matter [79,86,87,88,89]. DCHP and its metabolites can also be detected in human samples including urinary and blood samples, and some studies can detect high DCHP levels in certain blood samples (e.g., ~125 µg/L) [85,90,91,92,93]. Although humans can be exposed to high levels of DCHP, there are only a few studies to investigate DCHP’s adverse effects in animal models as compared with other well-studied phthalates such as DEHP. Our study demonstrated that chronic exposure to DCHP can increase atherosclerosis development in LDLR^−/−^ mice, and DCHP may have pro-atherogenic effects in humans. It is worth noting that the current study was conducted in mouse models and murine macrophages. PXR has been known to exhibit considerable pharmacological differences across mammalian species [20,94]. For example, we and others have reported that certain ligands (e.g., BPA) are potent agonists for human PXR, but not for mouse or rat PXR [2,21]. As for DCHP, we previously demonstrated that that DCHP can activate both human and mouse PXR with the similar efficacy in vitro [24]. Consistently, exposure to DCHP also led to activation of human PXR in PXR-humanized mice in vivo [24]. These results indicate that exposure to DCHP may also affect PXR signaling to have clinically relevant impact in humans.

In previous rodent studies, relatively high doses of DCHP (e.g., 10–2500 mg/kg/day) were used to treat rodents [95,96,97,98]. In addition, other phthalates such as DEHP have also been used at high doses (e.g., 1000 mg/kg BW/day) to treat mice in some studies [16,99]. The 10 mg/kg/day dosage we used for this study is lower than the concentrations used in most previous studies. We also considered the interspecies scaling factor of 12.3 between mice and humans, which reflects the 12.3-fold difference in surface area-to-body weight ratio between mice and humans [100,101,102]. Therefore, 12.3 times more chemicals are required to treat mice in order to obtain comparable doses used in humans [24,100,101]. We previously used similar doses for short-term exposure studies with PXR ligands, including phthalate substitute tributyl citrate (TBC) and several clinically used drugs [23,31,34,35]. The dose of 10 mg/kg/day for those ligands was able to efficiently activate PXR in vivo. Therefore, the 10 mg/kg/day dose was appropriate to study how chronic DCHP exposure affects PXR activity and atherosclerosis development in vivo.

In addition to PXR, some phthalates, including DEHP, have been shown to target other receptors, such as aryl hydrocarbon receptor (AhR) [103]. We previously investigated that ability of DCHP to activate other nuclear receptors and found that DCHP activated three forms of PXR including human, mouse, and rat PXRs, but did not activate other nuclear receptors including liver X receptor (LXR), farnesoid X receptor (FXR), and peroxisome proliferator-activated receptors (PPARs) [24]. However, we did not test AhR activity, so it is plausible that DCHP may affect other receptors or transcriptional factors to elicit the pro-atherogenic effects. In the current study, we found that that chronic exposure to DCHP increased atherosclerosis in PXR^F/F^LDLR^−/−^ mice, but not their PXR^ΔMye^LDLR^−/−^ littermates without affecting plasma lipid levels. Those littermates had the same PXR^F/F^LDLR^−/−^ double-mutant background, and PXR^ΔMye^LDLR^−/−^ mice carried heterozygous knock-in for LysM-Cre. Therefore, the increased atherosclerosis in PXR^F/F^LDLR^−/−^ mice exposed to DCHP are likely mediated through myeloid PXR signaling. It is intriguing that chronic DCHP exposure did not affect lipid levels in hyperlipidemic LDLR^−/−^ mice, as our recent study found that a short-term exposure (1 week) to the same dose of DCHP led to increased plasma cholesterol levels in wild type mice [24]. These results were likely due to the different genetic background of the mice as LDLR^−/−^ mice were already hyperlipidemic as compared with WT mice. It is possible the exposure to DCHP at the used dose was not sufficient to further enhance the already elevated plasma lipid level. Consistently, our previous study also found that exposure to a potent mouse PXR ligand, pregnenolone 16α-carbonitrile (PCN), induced hypercholesterolemia in WT mice but did not affect plasma total cholesterol levels in hyperlipidemic ApoE^−/−^ mice [26]. Further, chronic exposure to another PXR agonistic EDC, BPA increased atherosclerosis in PXR-humanized ApoE^−/−^ mice without altering plasma lipid levels. PXR can also be activated by microbially produced metabolites such as indoles and indole-derived metabolites in the gut [104,105,106]. Chronic activation of intestinal PXR by DCHP and those metabolites may also contribute the systemic effects of DCHP on myeloid functions and atherosclerosis development. Thus, it would be interesting to further investigate the impact of EDC- and microbial metabolite-mediated intestinal PXR activation on CVD risk in the future. 

Macrophages are one of major cell types contributing to the initiation, progression, and eventual rupture of atherosclerotic lesions [107,108], and macrophage accumulation within the vascular wall is a hallmark of atherosclerosis [43,109]. A family of scavenger receptors including CD36 and SRA are principal contributors to uptake of modified LDL such as oxLDL by macrophages [110,111,112]. In the current study, we found that DCHP exposure increased CD36 expression in macrophage of PXR^F/F^LDLR^−/−^ mice without significantly affecting the expression of other genes mediating macrophage lipid uptake and efflux including SRA, ABCA1, and ABCG1. While previous studies have also demonstrated that PXR does not regulate ABCA1 and ABCG1 expression in macrophages [25,26,32,45], DCHP treatment tended to increase ABCA1 and ABCG1 expression in microphages in the current study. It is plausible that chronic PXR activation may increase 4β-hydroxycholesterol levels, leading to activation of LXR that regulates ABCA1 and ABCG1 expression [36,113]. However, 4β-hydroxycholesterol-mediated LXR activation may not be strong enough to lead to significantly changed ABCA1 and ABCG1 expression in the current study. Future studies are required to investigate how the potential PXR-LXR crosstalk regulates key atherosclerosis-related gene expression in vitro and in vivo.

PXR has been shown to directly regulate CD36 transcription, and activation of PXR can promote CD36-meidated lipid accumulation in tissues [29]. We previously demonstrated that activation of PXR by other ligands including BPA and PCN increased CD36 expression and lipid accumulation in macrophages of ApoE^−/−^ mice in vivo, likely contributing to the increased atherosclerosis in those mice [26,32]. In the current study, DCHP-mediated PXR activation stimulated lipid uptake and foam cell formation in macrophages of PXR^F/F^LDLR^−/−^ mice. Chronic DCHP exposure also led to increased CD36 and macrophage content in the atherosclerotic lesions of PXR^F/F^LDLR^−/−^ mice, but not that of PXR^ΔMye^LDLR^−/−^ mice. Therefore, it is plausible that DCHP increased atherosclerosis in PXR^F/F^LDLR^−/−^ mice by stimulating CD36 expression and CD36-mediated lipid uptake and foam cell formation. Interestingly, Moreau et al. previously reported that PXR ligands did not affect CD36 expression in human hepatocytes [114]. It has been known that certain ligands can activate PXR and regulate its target gene expression in a tissue-specific manner [20,23,115,116]. While it is not clear whether PXR can regulate CD36 expression in human monocytes or macrophages, future studies are needed to investigate the detailed mechanisms through which PXR-agonistic EDCs regulate CD36 expression in human monocytes or macrophages to affect atherosclerosis development and CVD risk. 

In summary, we found that chronic exposure to a widely used phthalate, DCHP, increased atherosclerosis in LDLR^−/−^ mice in a myeloid PXR-dependent manner. DCHP exposure increased CD36 expression and foam cell formation in macrophages of PXR^F/F^LDLR^−/−^ mice, but deficiency of myeloid PXR inhibited DCHP-elicited macrophage dysfunction and atherosclerosis in PXR^ΔMye^LDLR^−/−^ mice. Our findings demonstrate, for the first time to our knowledge, that DCHP exposure increases atherosclerosis development in an appropriated laboratory animal model. These results may provide potential mechanisms underlying phthalates-associated CVD risk and may also stimulate further investigations of the adverse effects of plastic-associated EDCs on CVD risk in humans. 

## Data Availability

All data are included in figures in the manuscript and as Supplementary data.

## Supplementary Materials

The following are available online at https://www.mdpi.com/article/10.3390/cells11071125/s1, Table S1: Primer Sequences for QPCR.
