# Supplementary material for: Pregnane X Receptor Mediates Atherosclerosis Induced by Dicyclohexyl Phthalate in LDL Receptor-Deficient Mice
Source: Cells. 2022 Mar 26;11(7):1125. doi: 10.3390/cells11071125 (PMC8997706; doi:10.3390/cells11071125)
Supplement: Supplementary file 1 [file cells-11-01125-s001.zip › cells-1639695-supplementary.pdf]

**Supplemental Table S1. Primer Sequences for QPCR.**

| Name | Sequence |
|---|---|
| ABCA1 | 5’-CCGAGGAAGACGTGGACACCTTC-3’<br>5’-CCTCAGCCATGACCTGCCTTGTAG-3’ |
| ABCG1 | 5’-AGGTCTCAGCCTTCTAAAGTTCCTC-3’<br>5’-TCTCTCGAATGAAATTTATCG-3’ |
| 36B4 | 5’-CCAGGAAGGCCTTGACCTTT-3’<br>5’-CTGATCATCCAGCAGGTGTT-3’ |
| CD36 | 5’-CAGTCGGAGACATGCT-3’<br>5’-CTCGGGGTCCTGAGTT-3’ |
| GAPDH | 5’-AACTTTGGCATTGTGGAAGG-3’<br>5’-GGATGCAGGGATGATGTTCT-3’ |
| LOX1 | 5’-GCTGCAAACTTTTCAGGTCCT-3’<br>5’-AGGTGGTATGGGAAATTGCTTG-3’ |
| SR-A | 5’-GGAGTGTAGGCGGATC-3’<br>5’-GTCAATGGAGGCCCCA-3’ |
